# Microbe-set enrichment analysis facilitates functional interpretation of microbiome profiling data

**DOI:** 10.1038/s41598-020-78511-y

**Published:** 2020-12-08

**Authors:** Yan Kou, Xiaomin Xu, Zhengnong Zhu, Lei Dai, Yan Tan

**Affiliations:** 1 Xbiome, Scientific Research Building, Room 907, Tsinghua High-Tech Park, Shenzhen, China; 2 CAS Key Laboratory of Quantitative Engineering Biology, Shenzhen Institute of Synthetic Biology, Shenzhen Institutes of Advanced Technology, Chinese Academy of Sciences, Shenzhen, 518055 China

**Keywords:** Data integration, Data mining, Literature mining

## Abstract

The commensal microbiome is known to influence a variety of host phenotypes. Microbiome profiling followed by differential abundance analysis has been established as an effective approach to study the mechanisms of host-microbiome interactions. However, it is challenging to interpret the collective functions of the resultant microbe-sets due to the lack of well-organized functional characterization of commensal microbiome. We developed microbe-set enrichment analysis (MSEA) to enable the functional interpretation of microbe-sets by examining the statistical significance of their overlaps with annotated groups of microbes that share common attributes such as biological function or phylogenetic similarity. We then constructed microbe-set libraries by query PubMed to find microbe-mammalian gene associations and disease associations by parsing the Disbiome database. To demonstrate the utility of our novel MSEA methodology, we carried out three case studies using publicly available curated knowledge resource and microbiome profiling datasets focusing on human diseases. We found MSEA not only yields consistent findings with the original studies, but also recovers insights about disease mechanisms that are supported by the literature. Overall, MSEA is a useful knowledge-based computational approach to interpret the functions of microbes, which can be integrated with microbiome profiling pipelines to help reveal the underlying mechanism of host-microbiome interactions.

## Introduction

With the advance in sequencing technology and growing interest in human microbiota, microbiome profiling datasets are accumulating rapidly. Standard microbiome data analysis pipelines primarily aim to identify individual microbial taxa, or microbial communities with differential abundance between healthy and diseased hosts. Then, genomic and/or metabolic strategies are used to characterize individual microbial taxa to help interpret their mechanisms in the pathogenesis of many complex human diseases^1,2^. The host-microbiome interactions are conveyed either by alteration of sets of microbes or by their collective functions.

Microbes are able to affect host phenotypes through modulation of gene expression^3^ or cell signaling in relevant host cells/tissues. However, the regulatory mechanisms of how microbiomes influence host physiology are not clear. Some studies demonstrated such host-microbiome interactions could be achieved via microbial metabolites. For instance, the host immune system has been shown to be modulated by the gut microbiome via microbial metabolites^4,5^. As a component of the Human Functional Genomics Project (HFGP), Schirmer et al.^4^ found correlation between gut microbial features and production of various types of cytokines in a cohort of 500 healthy adults from the Netherlands. Next, they experimentally validated that two microbial metabolites, tryptophol and palmioleic acid, are able to modulate the production of IFNγ and TNFα, respectively, in peripheral blood mononuclear cells. In an in-depth investigation^5^, identified microbe-derived metabolite, ascorbate, as a selective inhibitor of activated CD4+ effector T cells, including IL-17A-, IL-4-, and IFNγ-producing cells. However, these mechanistic studies are resource intensive and often prone to empirical biases.

As the knowledge about differential abundance of human microbiome species between healthy and diseases accumulates with the surge of microbiome profiling studies, our understanding of the mechanisms of how microbiome influence human phenotypes are still limited because of the complexity of host-microbe interactions. There is an urgent need for bioinformatics tools that leverage curated and structured knowledge to guide experimental studies. Functional enrichment analysis for gene-centric data, such as transcriptomics and proteomics, helps interpret sets of differentially expressed genes through prior knowledge about gene functions^6^. Similarly, it would be enormously useful to organize the knowledge about the effects of microbes on the host to aid the functional interpretation of microbiome datasets/signatures. Microbes can be grouped into microbe-sets based on shared attributes. Themed collections of such microbe-sets can be organized into microbe-set library as a representation of knowledge.

Recently, an increasing number of such resources have been established. Disbiome^7^ emerged as the first database cataloging microbial composition differences in diseases, which covers 190 human diseases, 800 microbial organisms across 674 published studies. There are also databases categorizing microbes based on genomic^8^, protein family^9^ and taxonomic information^10^. In addition, the research community established databases documenting different functional aspects of microbes including pathogenesis (e.g. EuPathDB^11^), transport and metabolism (e.g. TCDB^12^) and signal transduction and gene regulation (e.g. MiST^13^). These databases are valuable for deciphering the molecular mechanisms of how microbes influence host phenotypes. However, the cumulative knowledge on mechanistic studies of microbes and diseases is often scattered in literature.

In this study, we developed microbe-set enrichment analysis (MSEA), a novel computational approach for interpreting microbe-sets using themed collections of functionally annotated microbe-sets representing prior knowledge. We demonstrated the outstanding utility of the MSEA methodology by carried out three case studies using publicly available curated knowledge resource and microbiome profiling datasets focusing on human diseases. We found MSEA not only yields consistent findings with the original studies, but also uncovers insights about disease mechanisms that are supported in the literatures. To disseminate our method to the microbiome research community, we developed a Python package “msea” to enable investigators to adopt this analytical approach (available at https://pypi.org/project/msea/).

## Results

### Construction of a microbe-set library from PubMed literature

**Figure 1:**
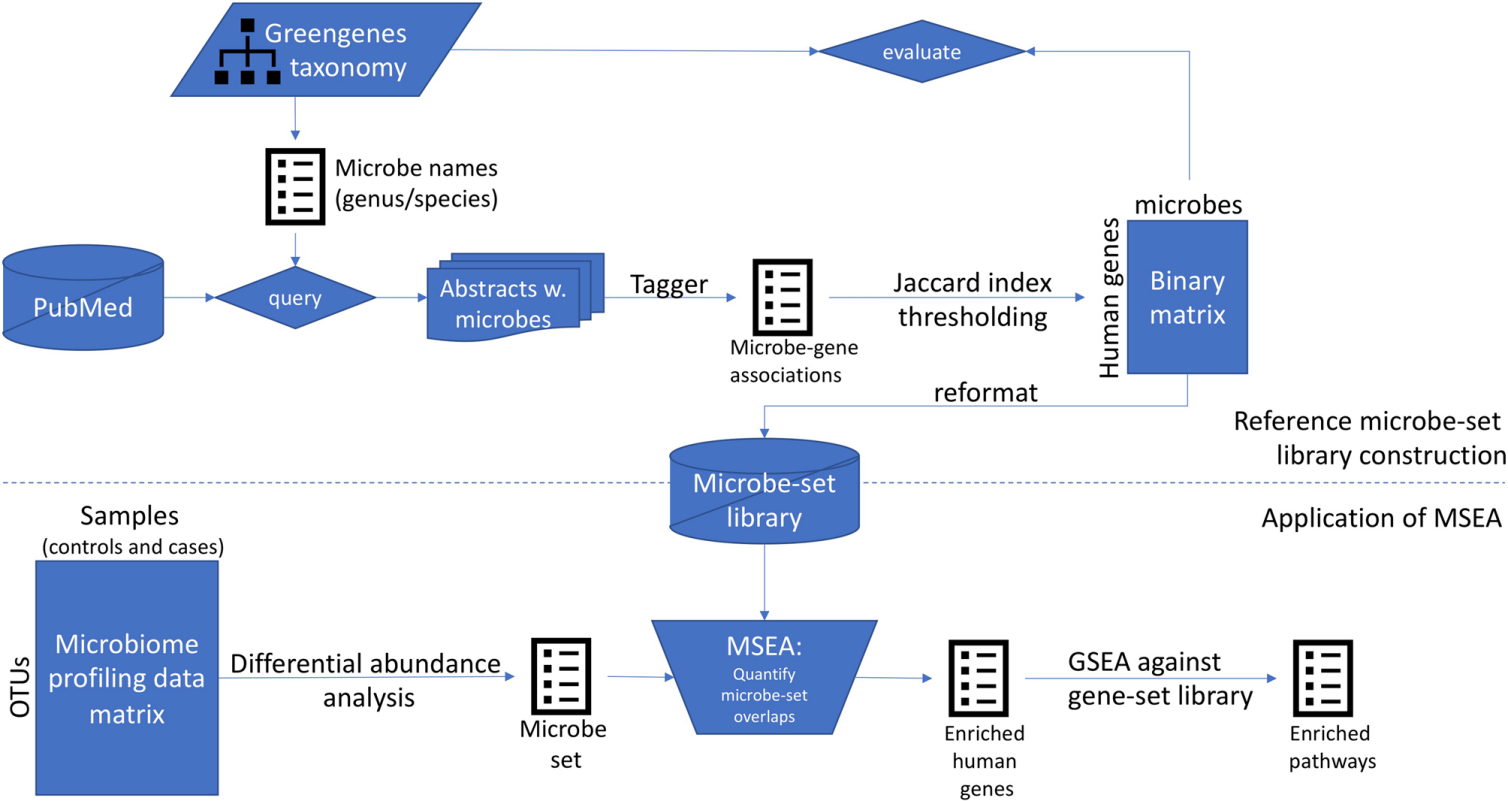
Chart showing the workflow of the construction of microbe-set library and application of MSEA.

Enrichment analysis is designed to infer the collective functions for a set of microbes instead of individual ones by identifying microbe-sets sharing common attributes with the input microbe-set. To perform MSEA, we first created a microbe-set library from PubMed literature as the background knowledge representation (Fig. 1). Since we aim to study the host-microbiome interactions to investigate how gut microbial organisms affect host phenotypes via the expression of host genes, we grouped microbes based on their literature-documented associations with mammalian genes. The microbe-gene associations were defined as significant co-occurrence across millions of PubMed abstracts. To create this comprehensive collection of literature-based microbe-gene associations, we first parsed the taxonomy information from Greengenes^14^ to get 1085 microbial genus and species names across *Bacteria* and *Archaea* kingdoms. The names of those microbial species were then used as search terms to query PubMed via Geneshot^15^. Amongst 978,217 PubMed abstracts hits across the 1085 queries, 970 microbial names returned at least one PubMed hits, mentions of 8865 distinct mammalian genes were recognized and mapped to HUGO Gene Nomenclature Committee (HGNC) gene symbols by the named-entity recognition (NER) tool Tagger^16^. We next computed Jaccard Index to quantify the association strength between microbe and mammalian genes to filter out week associations that were observed by chance. The filtering led to 42,944 associations covering 752 microbes and 2045 mammalian genes.

As expected, mammalian genes that are most frequently associated with microbial entities are related to immunity and inflammatory responses, such as genes encoding cytokines including *TNF*, *IL10* and *IL6*, as well as genes involved in innate immune responses such as Toll-like receptors (TLRs) and innate immune signal transduction adaptor *MYD88* (Table 1).

**Table 1:** Mammalian genes with most microbe-gene associations from PubMed literature.

Mammalian gene	HGNC symbol	Microbe count
Tumor Necrosis Factor	*TNF*	401
Interleukin 10	*IL10*	278
Toll Like Receptor 4	*TLR4*	263
Toll Like Receptor 2	*TLR2*	238
Fos Proto-Oncogene	*FOS*	213
Angiotensin I Converting Enzyme	*ACE*	209
C-Reactive Protein	*CRP*	203
Caspase 3	*CASP3*	182
Myeloid Differentiation Primary Response 88	*MYD88*	182
Glyceraldehyde-3-Phosphate Dehydrogenase	*GAPDH*	168
Interleukin 6	*IL6*	160
Prostaglandin-Endoperoxide Synthase 2	*PTGS2*	157
Forkhead Box P3	*FOXP3*	155
C–C Motif Chemokine Ligand 2	*CCL2*	144
CD86 Antigen	*CD86*	139
Nucleotide Binding Oligomerization Domain Containing 2	*NOD2*	139
Caspase 1	*CASP1*	132
Toll Like Receptor 9	*TLR9*	128
CD40 Antigen	*CD40*	125
Intercellular Adhesion Molecule 1	*ICAM1*	124

Interestingly, genes without apparent roles in immunity such as proto-oncogene *FOS* and apoptosis-related cysteine protease *CASP3* are also shown to have many microbial associations. FOS is a central transcriptional regulator for innate immune system^17^. CASP3, although serves its function canonically in apoptosis, is also involved in inflammatory response and B-cell activation ^18^. We also found the top microbial genus and species with the most mammalian gene associations includes well-characterized microbial species used as model organisms (e.g. *Escherichia coli* and *Saccharomyces cerevisiae*), highly common commensal bacterium (e.g. *Staphylococcus aureus* and *Lactobacillus*) and certain well-known pathogens (e.g. *Salmonella enterica*, *Pseudomonas aeruginosa* and *Helicobacter pylori*) (Table 2).

**Table 2:** Microbial genus and species with most mammalian gene associations from PubMed publications.

Microbe	Gene count
*Escherichia*	959
*Escherichia coli*	957
*Enterobacteriaceae bacterium*	952
*Streptococcus *sp*.*	858
*Streptococcus*	858
*Pseudomonas*	799
*Staphylococcus*	796
*Staphylococcus aureus*	788
*Bacillus*	785
*Aerococcus viridans*	757
*Mycobacterium*	739
*Epsilonproteobacteria*	705
*Pseudomonas aeruginosa*	688
*Helicobacter pylori*	665
*Salmonella enterica*	649
*Saccharomyces*	616
*Saccharomyces cerevisiae*	614
*Lactobacillus*	606
*Clostridium*	594
*Alphaproteobacteria*	544

**Figure 2:**
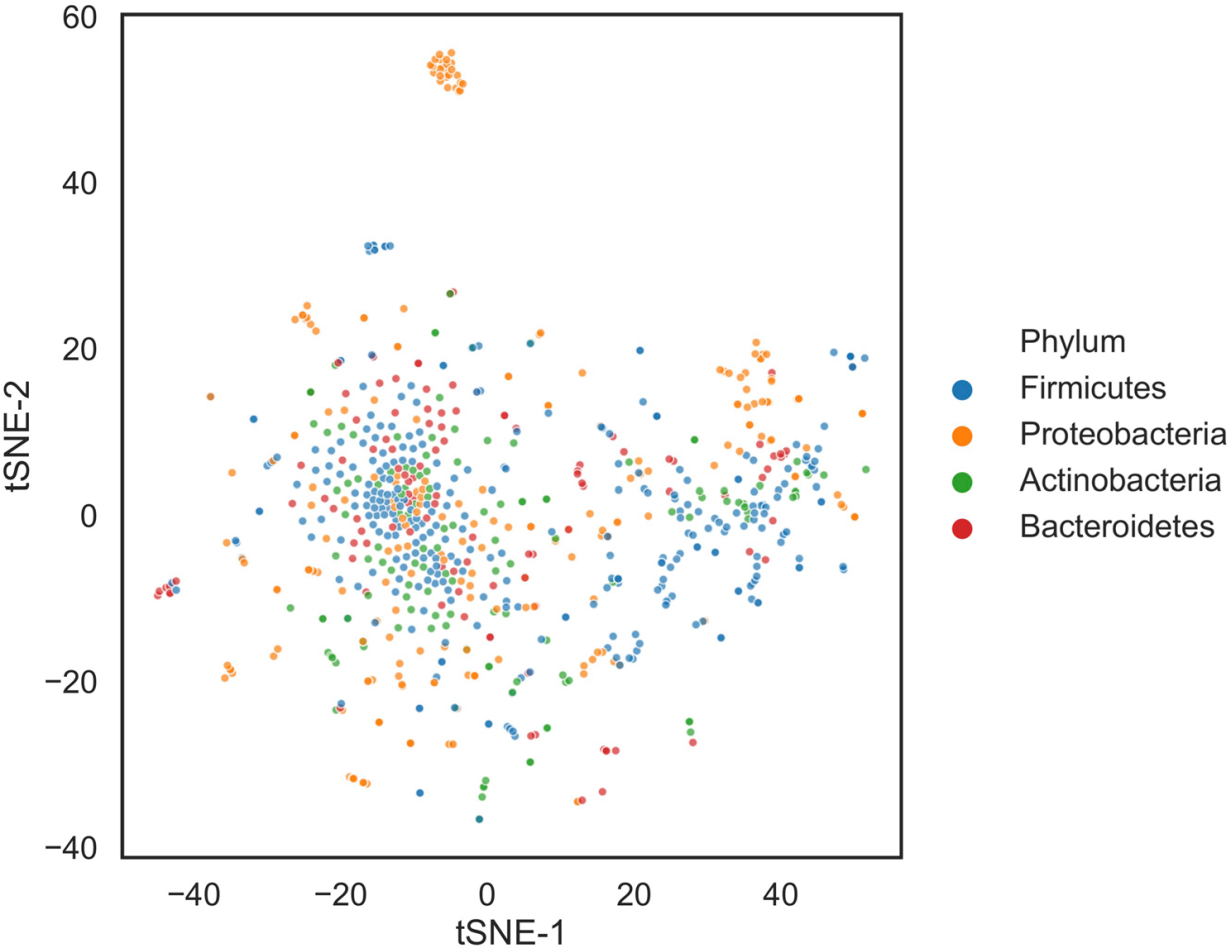
t-SNE visualization of the normalized microbe-gene co-mentioning matrix derived from PubMed queries for the microbes from the following four phyla: *Firmicutes*, *Proteobacteria*, *Bacteroidetes*, and *Actinobacteria*. The t-SNE was applied to the TF-IDF normalized (see “Methods”) microbe-gene co-mentioning matrix to calculate the 2-D coordinates for individual microbial genus or species. Each dot in the scatter plot represents a microbial genus or species, which is colored by their respective phylum based on Greengenes taxonomy.

To globally assess the quality of the microbe-gene associations constructed from PubMed abstracts, we intersected the microbe-gene associations with an independent and objective knowledge resource, the taxonomy for microbes from Greengenes^14^. The assumption for this assessment is that the set of microbes associated with the same mammalian genes are more likely to be enriched among certain taxonomic clades than random. We reduced the dimensionality of the microbe-set library of microbe-gene associations using t-Distributed Stochastic Neighbor Embedding (t-SNE)^19^ to derive an embedding for microbial genus and species based on their potential functional association spectrum of mammalian genes (Fig. 2). By overlaying the phylum information onto the t-SNE embedding, we observed several clusters of microbes including *Firmicutes* and *Proteobateria* belong to the same phylum. These results validated our approach of automated curating microbe-gene associations from the literature is able to recapitulate, to some extent, phylogenic similarities among microbes. The resultant microbe-set library also lays the foundation of subsequent case studies of MSEA.

To demonstrate the use cases and effectiveness of our newly devised MSEA methodology, we carried out the following three case studies with real-word microbiome datasets from diverse biological contexts.

### Case study 1: MSEA between disease-centric microbe-sets and gene-centric microbe-sets

First, we set up a case study for MSEA to examine whether microbe-sets can be used as an intermediary to connect mammalian genes and diseases. The rationale behind the case study is that many microbes are observed to associate with a variety of human diseases, we argue some of those links could be due to their ability to regulate certain mammalian genes that are implicated in the diseases. Therefore, one would expect to find known gene-disease associations via MSEA between disease-centric microbe-sets and gene-centric microbe-sets. To construct microbe-set library associating human diseases with microbes, we used the Disbiome database^7^, a resource for microbiome composition differences in diseases curated from case–control studies. On the other hand, we used the microbe-sets library of microbe- mammalian gene associations from literature as the background.

MSEA analysis found several interesting microbe-mediated disease-gene associations (Table 3). For example, microbes with differential abundance in non-alcoholic fatty liver disease (NAFLD) are significantly enriched for *SREBF1* and *LPL* genes via literature-based associations (Table 3). *SREBF1*, which encodes a sterol regulatory element binding transcription factor, is the known regulator of cholesterol and fatty acid synthesis in the liver^20^. Overexpression of *SREBF1* was also shown to cause NAFLD in mice^21^. Additionally, lipoprotein lipase (*LPL*) also has well-characterized role in the pathophysiology of NAFLD: lipoprotein metabolism is the central pathway for the hepatocellular lipid homeostasis^22,23^; more recently, the up-regulation of *LPL* in hepatic stellate cells has also been demonstrated to exacerbate liver fibrosis in non-alcoholic steatohepatitis (NASH)^24^, which can be considered as a subtype of NAFLD. Hence, the roles of those overlapping microbes associated with *SREBF1* and *LPL* that also exhibit abnormal abundance in non-alcoholic fatty liver disease merit further investigations.

**Table 3:** Top enriched gene-disease connections via microbes identified using MSEA.

Gene	Disease	Odds ratio	P-value	q-value	Combined score (see “Methods”)	Supporting references	Shared microbes
SREBF1	Non-alcoholic fatty liver disease	12.2	9.8E−08	2.0E−04	63.80	^20,21^	11
LPL	Non-alcoholic fatty liver disease	18.4	2.8E−07	2.9E−04	20.43	^22–24^	8
FABP4	Hepatitis C	14.7	2.6E−07	5.4E−04	− 1.07	^25^	9
ATG16L1	Crohn's disease	3.9	2.2E−06	1.6E−03	1.65	^26^	23
CCL11	Crohn's disease	3.6	1.2E−06	1.6E−03	− 14.42	^27,28^	27
FUT2	Crohn's disease	4.0	1.7E−06	1.6E−03	30.03	^29,30^	23
HCK	Asthma	14.6	4.9E−06	1.7E−03	22.60	^31^	7
RAB14	Asthma	15.9	3.1E−06	1.7E−03	− 12.40	^32^	7
TF	Asthma	8.7	1.1E−05	1.7E−03	− 14.60	^33^	9
MAPK8	Asthma	9.8	4.8E−06	1.7E−03	18.64	^34^	9

Another notable results from the MSEA found microbes with differential abundance in Crohn’s disease are enriched for microbes associated with *ATG16L1*, *CCL11* and *FUT2* (Table 3), all of which have been implicated in the pathology of Crohn’s diseases, an inflammatory bowel disease (IBD). Specifically, *CCL11*, an eosinophil-specific chemokine, is significantly elevated in serum of Crohn’s disease patients versus normal controls^27^ and has been shown to be a central mediator for eosinophil recruitment in colon^28^. The genetic polymorphisms of Fucosyltransferase 2 (*FUT2*) have also been associated with Crohn’s diseases in multiple independent genome-wide association studies from distinctive populations^29,30^. The role of Autophagy Related 16 Like 1 (*ATG16L1*) in Crohn’s diseases is even more well-characterized by a large body of literature (See^26^ for a comprehensive review).

It is encouraging that our MSEA approach crossing gene-microbe associations and disease-microbe associations is able to recover some known relationships between mammalian genes and human diseases, including NAFLD and IBD. These observations lend support to the hypothesis that host-associated microbiome plays an important role in a variety of diseases.

Next, we present additional case studies that use MSEA and microbiome profiling (16S rRNA sequencing data from patients or animal models) to study the role of microbiome in two specific diseases.

### Case study 2: MSEA uncovers microglia activation by the gut microbiota of Parkinson’s disease patients

In a study published in 2016^35^, Sampson and colleagues revealed the functional connections between gut microbiota and the pathology of Parkinson’s disease (PD): gut microbes promote α-synuclein-mediated motor deficits and microglia activation in mouse brains. More specifically, the gut microbes are able to modulate microglia and enhance PD pathophysiology through production of microbial metabolites short-chain fatty acids (SCFAs). In their experiments, Sampson and colleges performed fecal transplant from PD and healthy human donors to germ-free wild-type mice and mice overexpressing alpha-synuclein, then carried out microbiome profiling using 16S rRNA sequencing. Their resulting microbiome data only revealed distinctive compositions of microbial communities between PD and health donors persist in mice. Their finding on the connection between PD-specific microbiota and microglia activation was reached via a series of more sophisticated experiments in mice including immunostaining, ELISA and qPCR to identify microglia-specific marker genes.

**Figure 3:**
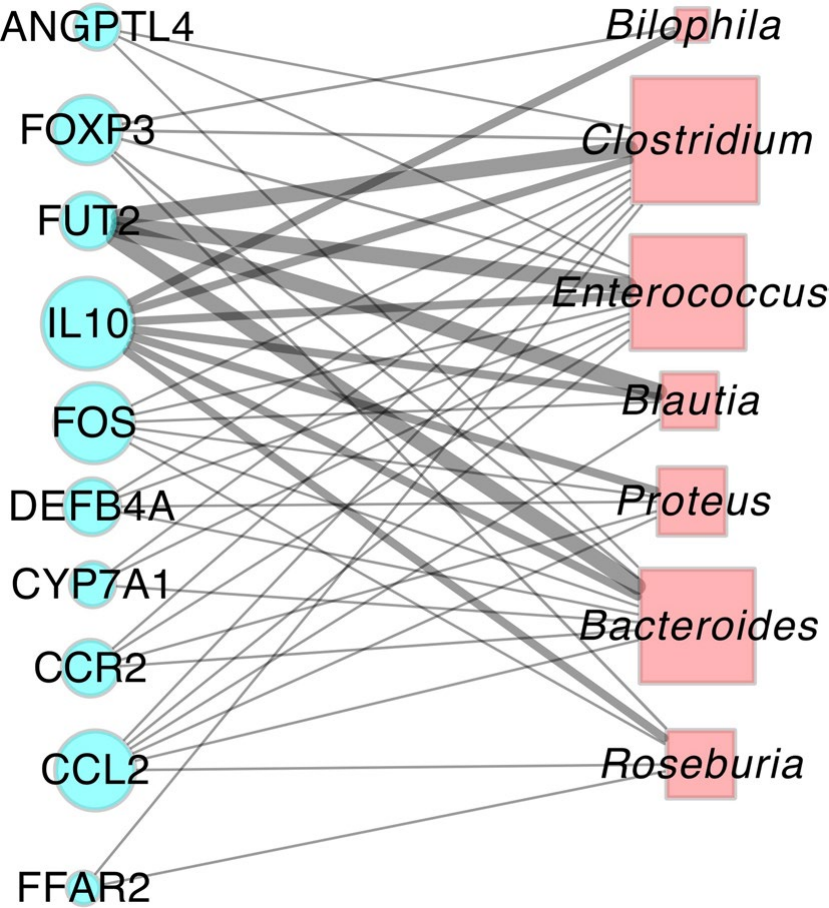
Bipartite graph visualizing the enriched mammalian genes with their associated DA microbes in PD versus healthy controls. Genes are charted as blue round nodes whereas DA microbes are plotted as red squares. The sizes of the nodes are proportional to the number of edges in the bipartite graph whereas the width of the edges indicates the strength of the enrichment measured by combined scores from the MSEA algorithm.

In this case study, we re-analyzed their 16S microbiome profiling data with a focus on characterizing the functions of microbes with differential abundance (DA) in PD compared to healthy controls using MSEA. We first downloaded the 16S dataset from Qiita^36^ (Study ID: 10483). Next, by applying ANCOM^37^, we were able to reproduce the DA microbes in mice transplanted with fecal samples from PD donors reported in the original study (Fig. S2). With these DA microbes as input, we next applied MSEA to prioritize mammalian genes enriched for those PD-related DA microbes based on literature-based associations. Among the top enriched genes (Fig. 3; Table S2), we found some immune-related genes such as *IL10*, *FOXP3*, *DEFB4A*, *CCL2* and *CCR2*. This suggests DA microbes in PD can potentially affect the immune system by modulating those genes involved in various types of immune responses, which is consistent with the finding in the original study that gut microbiota impact neuroimmune responses in a mouse model of PD. We also checked the enrichment of two pro-inflammatory cytokines with elevated expression in the brains of PD patients, including tumor necrosis factor-α (*TNF-α*) and interleukin-6 (*IL-6*) to find that *IL-6* are also marginally enriched in the DA microbes (p-value = 9.7e−3; combined score = 4.42). This result suggests that the microbes with altered abundance in PD may be able to up-regulate the pro-inflammatory cytokines such as *IL-6* to induce the neuroinflammation state of PD.

We next asked what are the collective functions of the top enriched genes from DA microbes in PD versus healthy controls to shed lights on their potential roles on neuroinflammation. To do that, we performed gene function enrichment analysis using Enrichr^38^ to reveal these genes are significantly enriched for microglia-associated genes (p-value = 5.8e−6), which is in agreement with the finding in the original study that gut microbiota regulates microglia activation.

In our re-analysis of the dataset, we were able to apply MSEA to the DA microbes in PD to find enriched mammalian genes, many of which are immune-related, indicating the significant role of gut microbiome in neuroimmune responses. Further gene function enrichment analysis found many of those mammalian genes are involved in microglia development, consistent with the ultimate finding from the original study based on extensive animal experiments. This case study demonstrates that MSEA is a powerful tool for revealing the hidden signals in microbiome profiling data.

### Case study 3: MSEA identifies immune response pathways associated with the gut microbiome in a DSS-induced colitis mouse model

In this case study, we reanalyzed a microbiome profiling dataset from a study published in 2018^39^, where Nunberg and colleagues used a Dextran Sodium Sulfate (DSS)-induced colitis mouse model to study the connection between gut microbiota dysbiosis and patterns of IBD development. They also demonstrated how IL-1α deficiency confers a protective effect in DSS-induced colitis via altered gut microbiota composition.

We first downloaded the 16S dataset from Qiita^36^ (Study ID: 11123). The dataset covers 221 mouse samples from WT (wildtype) and IL-1α knockout (KO) mice at 0, 1, 8 and 14 days after DSS administration. Consistent with the findings in the original publication, we observed that fecal samples from IL-1α KO mice without co-housing with other mice exhibit the most distinctive microbiome profile (Fig. S3). We also reproduced the top DA microbial genera between IL-1α KO and WT mice, including *Bacteroides, Akkermansia* and *Turicibacter* (Fig. S4). Next, we seek to interpret the functions of those DA microbes using MSEA to shed light on the mechanism of colitis-resistance for the IL-1α-deficient mice.

**Figure 4:**
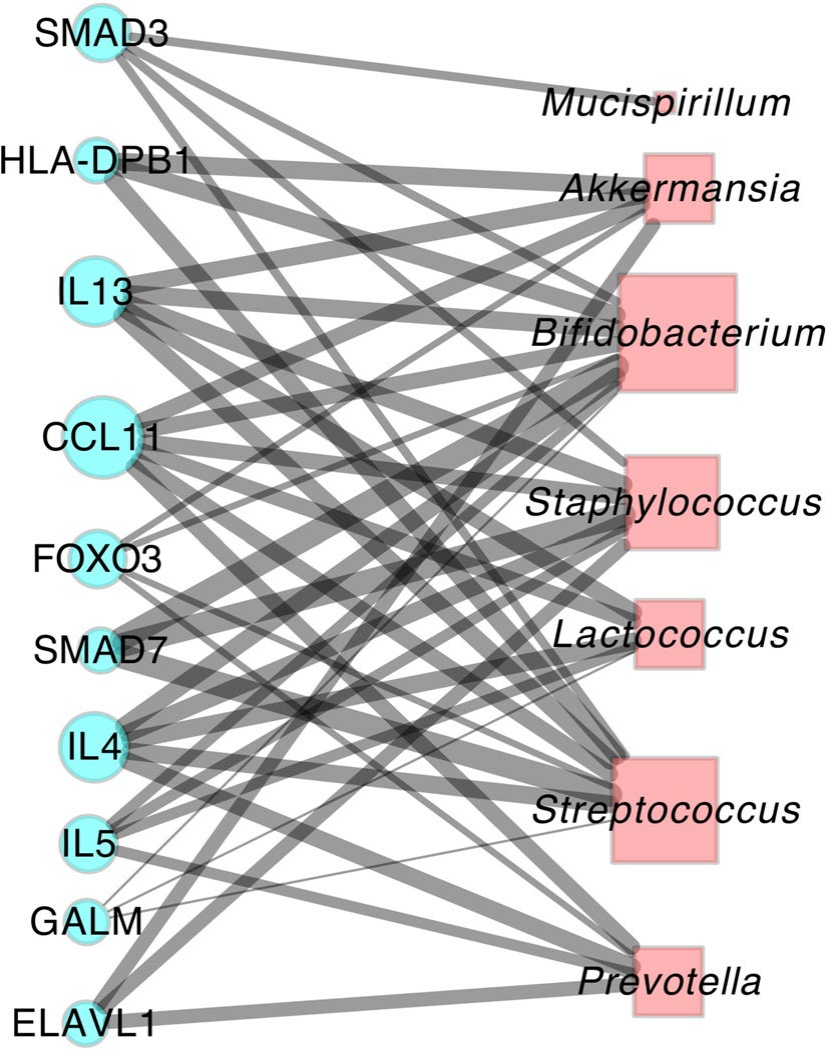
Bipartite graph visualizing the enriched mammalian genes with their associated DA microbes in IL-1α-KO versus WT mice 8 days after DSS-administration. Genes are charted as blue round nodes whereas DA microbes are plotted as red squares. The sizes of the nodes are proportional to the degree in the bipartite graph whereas the width of the edges indicates the strength of the enrichment measured by combined scores from the MSEA algorithm.

We found that DA microbes between IL-1α-deficient and WT mice on day 8 after DSS-treatment are significantly enriched for many interleukins (ILs), including IL13, IL4 and IL5 (Fig. 4; Table S3), suggesting some of the DA microbes in response to IL-1α-deficiency may be associated with the productions of those other ILs, which forms a cascade to de-sensitize the inflammatory response in the gut, thus making it less prone to IBD such as colitis.

**Figure 5:**
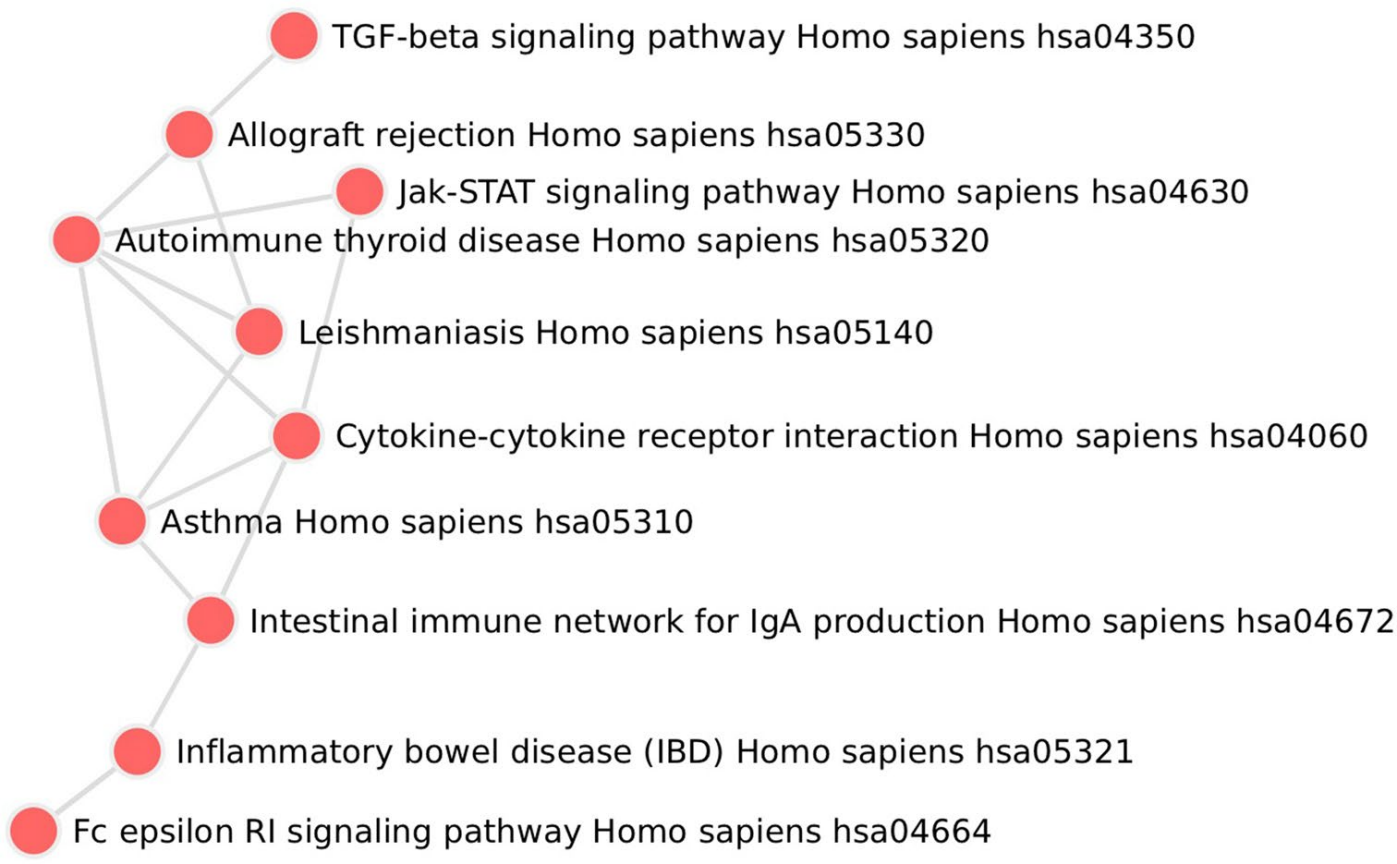
Network of top enriched human KEGG pathways for genes enriched from MSEA analysis using DA microbes in IL-1α-KO versus WT mice 8 days after DSS-administration. Nodes in the network are enriched human KEGG pathways. The pathways are connected if they have significant number of overlapping genes determined by Fisher’s exact test.

We next performed enrichment analysis for the top genes that are enriched from the DA microbes on day 8 to find they are enriched for the genes known to be involved in the pathogenesis of IBD curated in KEGG pathway, along with other pathways essential for immune responses such as Jak-STAT signaling pathway, cytokine-cytokine receptor interaction as well as immune dysregulation disorders such as asthma and autoimmune thyroid disease (Table 4, Fig. 5). These finding provides more evidence on how the DA microbes in IL-1α-deficient mice are possibly able to modulate the host immune response via acting on cytokines and other key pathways.

**Table 4:** Top enriched human KEGG pathways for genes enriched from MSEA analysis using DA microbes in IL-1α-KO versus WT mice 8 days after DSS-administration.

Rank	Term	P-value	q-value	Z-score	Combined score
1	Asthma *Homo sapiens* hsa05310	1.6E−12	4.7E−10	322.6	8762.2
2	Inflammatory bowel disease (IBD) *Homo sapiens* hsa05321	7.7E−11	1.1E−08	153.8	3582.4
3	Allograft rejection *Homo sapiens* hsa05330	7.5E−07	7.3E−05	157.9	2226.3
4	Intestinal immune network for IgA production *Homo sapiens* hsa04672	1.5E−06	1.1E−04	125.0	1673.1
5	Autoimmune thyroid disease *Homo sapiens* hsa05320	2.1E−06	1.2E−04	113.2	1481.1
6	Fc epsilon RI signaling pathway *Homo sapiens* hsa04664	4.4E−06	2.2E−04	88.2	1087.6
7	Cytokine-cytokine receptor interaction *Homo sapiens* hsa04060	5.9E−06	2.5E−04	30.2	363.3
8	Jak-STAT signaling pathway *Homo sapiens* hsa04630	5.6E−05	2.0E−03	38.0	372.0
9	Leishmaniasis *Homo sapiens* hsa05140	5.8E−04	1.9E−02	54.8	408.3
10	TGF-beta signaling pathway *Homo sapiens* hsa04350	7.7E−04	2.2E−02	47.6	341.5

In this case study, our reanalysis of the dataset in a DSS-induced colitis mouse model identified specific interleukins and other genes associated with the altered gut microbiota in the pathogenesis of IBD. We demonstrated that MSEA is an excellent tool to interpret the potential functions of DA microbes from microbiome profiling datasets, thus helping experimental microbiologists to generate testable hypothesis and providing mechanistic insights on how changes in microbiota composition influence the expression of host genes.

## Discussion

Interpreting the functions of microbe sets from microbiome profiling experiments is central to discovering potentially novel underlying host-microbial interactions. Here we developed the MSEA methodology to fulfill exactly this purpose: taking any microbe sets as input, then statistically examining the overlaps with annotated microbe-sets to prioritize enriched functions. In the three case studies demonstrated, although we only used annotated microbe sets associated to mammalian genes based on literature evidences, MSEA is versatile and can be expanded to other types of annotated microbe sets. As more functional microbiome datasets become publicly available, one could organize microbe-sets by their shared experimental conditions or treatment. For instance, one approach is to collect microbes with differential abundance after drug treatment to compile a microbe-set library for drugs^40,41^. Microbe sets can also be constructed as fuzzy sets, where the membership between a microbe-set and individual microbe can be partial. The partial membership allows a more quantitative representation of the associations between a microbe and a functional term.

Microbiome datasets and resources are still limited compared to large-scale omics datasets and knowledge resources about mammalian genes. One advantage of MSEA is the ability to port microbe sets to mammalian gene sets via gene-microbe associations. Once MSEA finds a list of mammalian genes enriched within the input microbe set, microbiologists can use the mammalian genes as input for GSEA (gene-set enrichment analysis), which opens access to a variety of gene-set libraries representing human pathways, ontologies, phenotypes, diseases and cellular and tissue contexts.

However, the proposed MSEA is not without limitations. First of all, it doesn’t take the confidence of identifying a microbial species from a microbiome profiling dataset into consideration. This might lead to inaccurate results stemmed from OTUs (operational taxonomic units) misclassification to microbial species. Secondly, MSEA currently treats all microbes in an input set equally, which might not necessary be the case for experimentally derived microbe-sets: some microbes may have larger effect sizes and statistical significance for enrichment between experimental group and control groups. Future enhancement would take the weightings of microbes in an input microbe-set into account to potentially improve the results of functional prioritization. Thirdly, we constructed gene-microbe associations from text-mining of PubMed abstracts, which could be not ideal in terms of qualities compared to extracting such associations from gene expression profiles of gnotobiotic mice colonized by single microbial species. However, those types of approaches require nontrivial amount of manual dataset curations and re-analyses, underscoring the value of curated knowledge and databases such as Disbiome ^7^. Lastly, we plan to develop a web-based interface for the “msea” software in the future, which would lower the barrier of entries for researchers.

## Methods

### Construction of microbe-set libraries through taxonomy and PubMed queries

We constructed themed collections of microbe-sets, also known as microbe-set libraries as a way to represent knowledge about individual microbial species or other taxonomic levels. In this study, we constructed microbe-set libraries through taxonomy and PubMed literatures.

To compile the taxonomy-based microbe-set library, we downloaded the Greengenes database release 13_5^14^ from^42^. Next, a phylogenetic tree was constructed as a directed acyclic graph (DAG) to include all existing taxonomic units in the Greengenes database up to species level (excluding strains) as nodes of the DAG. Two kingdoms, *Viruses* and *Viroids*, were excluded from the tree because the datasets of interest in this study came primarily from 16S, which do not contain any viruses. We also normalized the strings used to describe the microbes in Greengenes by removing the suffixes including “_noname” and “_unclassified”. We then constructed taxonomy-based microbe-set library by converting the phylogenetic tree to collection of microbe-sets by enumerating all leaf nodes from the parent nodes at a certain taxonomic rank (e.g. *Order*, *Family*). This taxonomy-based microbe-set library represents phylogenetic similarity among microbial taxonomic units.

The purpose of compiling literature-based microbe-set library is to group together microbes with similar functional associations to mammalian genes. To do that, we first queried the normalized microbe names from the Greengene taxonomy at the genus- and species- levels against PubMed abstracts using Geneshot^15^, which returns a list of PubMed abstracts with the query as well as automatically recognized mentions of mammalian genes using the named-entity recognition (NER) tool Tagger^16^. This procedure provided microbe-gene associations across a number of PubMed abstracts.

For visualization purpose, we normalized the microbe by gene matrix using term frequency-inverse document frequency (TF-IDF), which reflects the relative importance of microbes with respect to genes while offsetting the overall frequency of microbes across the corpus of the retrieved PubMed abstracts.

To construct microbe-set library based on the co-occurrence from literatures, we first quantified the association strength between a pair of microbe and human gene, we adopted Jaccard Index defined as:

$$\frac{{\left| {{\rm{abstracts}}\;{\rm{co - mentioning}}\;microb{e_i},\;gen{e_j}} \right|}}{{|{\rm{abstracts}}\;{\rm{mentioning}}\;microb{e_i}| + |{\rm{abstracts}}\;{\rm{mentioning}}\;gen{e_j}| - |{\rm{abstracts}}\;{\rm{co - mentioning}}\;microb{e_i},\;gen{e_j}|}}$$

The Jaccard Index effectively quantifies the association strength of a pair of microbe and human gene both mentioned in a particular article over such association is observed simply by chance. Next, we applied threshold for the Jaccard Index of 0.0028, which corresponds to top 0.1% of all the possible pairs, to binarize microbe-gene associations. The threshold for Jaccard Index was selected to optimize the overall correlation between the taxonomy tree and the microbe-gene associations (Fig. S5). The collection of microbe-gene pairs was then converted to microbe-set library by organizing microbes into sets by their shared mammalian genes with significant association based on literature co-mentioning.

### Statistical procedures for enrichment analyses of microbe-sets

Similar to bioinformatics enrichment analyses for genes, enrichment for microbe-sets can also be broadly classified into singular enrichment analysis (SEA); gene set enrichment analysis (GSEA); and modular enrichment analysis (MEA)^43^. In this study, we primarily adopted the traditional strategy of SEA for microbe-set enrichment analysis (MSEA). The SEA strategy takes a pre-defined set of microbes as input, and then iteratively test the enrichment of each annotated background microbe-sets independently. Afterwards, the individual, enriched curated microbe-sets passing the enrichment score threshold are reported in a tabular format as ordered by the statistical confidence of the overlap between the input microbe-set and the curated microbe-set to suggest pertinent microbial functional interpretations.

To quantitatively measure the overlap between two microbe-sets, we used Fisher’s exact test, which assumes a binomial distribution and independence for probability of any microbes belonging to any set. The universe size for the Fisher’s exact test used throughout the MSEA analyses in this manuscript was set to 1000, which is determined by the microbiome profiling pipeline as well as the reference microbe-set library.

Concretely, the microbe universe should be the union of all microbial species/genus the microbiome profiling pipeline can possibly identify and unique microbial species/genus in the microbe-set library. The number of microbial species/genus that a microbiome profiling pipeline could possibly identify is determined by (1) the experimental technology (16S or metagenomics, sequencing depth) and (2) the computational processing pipeline, specifically, the reference microbiome genomes.

All the case studies we performed in this manuscript applied Greenegenes taxonomy, which covers ~ 1000 distinct microbial species. On the other hand, the number of unique microbes in a microbe-set library depends on how the library was created. For instance, we primarily used the genus and species names from the Greengenes taxonomy to query PubMed to construct such as microbe-set library. The Greengenes taxonomy (version 13_5) covers 1085 distinct genera. Our resultant microbe-set library should ideally covers the same number of microbes, however, 115 genera were not associated with any PubMed hits, leading to 970 distinct genera in our library. Since the same taxonomy was used for both the microbiome profiling and the microbe-set library construction, the union of the two is the size of the Greengenes genus, which is ~ 1000.

It has been shown that Fisher’s exact test and related proportion tests, including Chi-square test and hypergeometric test, have some bias towards large set sizes. To correct for such bias, we adopted the procedure used in the gene set enrichment analysis tool Enrichr^38^. This procedure essentially used the expected rank for each curated gene-set with random input genes to correct for the observed rank. Briefly, we randomly sampled, without replacement, a universe of microbes under consideration. The random microbe-sets were then used for computing enrichment using Fisher’s exact test to estimate the expected ranks for the annotated microbe-sets. This procedure was repeated 10,000 times to compute the averages and standard deviations of the ranks for each annotated microbe-sets to compute a z-score for any future observed ranks from real microbe-set inputs. Alternatively, we also combined the p-value from Fisher’s exact test and the z-score measuring the deviation in expected ranks by multiplying these two numbers as follows:

$$c={\mathrm{log}}_{10}(p)\cdot  z,$$

to derive a combined score $c$.

The combined score has been shown to slightly outperform p-values in Fisher’s exact test and Z-score in identifying expected enrichment terms for differentially expressed genes from transcriptomics data^38,44^. However, due to a lack of microbiome datasets with biologically expected enriched mammalian genes, we were unable to confirm if this holds true in MSEA.

### Constructing microbe-set library from the Disbiome dataset

We exported the associations between human disease and microbes curated from publications from the Disbiome database^7^. Disbiome^7^ curated microbiome composition differences in diseases from case–control studies. For each curated study, Disbiome annotates the microbes that are elevated or reduced for the disease of interest, as well as experimental methods used for microbiome profiling. To construct a microbe-set library for human disease associtations, we parsed through 296 publications and collected microbes reported with differential abundances in diseased versus normal controls, regardless their directions (either elevated or reduced). We then grouped microbes associated with those diseases into microbe-sets. Collectively, we constructed a disease-centric microbe-set library covering 175 distinct human diseases, 755 microbial species and 2,760 disease-microbe connections with literature evidence.

### Reanalysis of the Parkinson’s disease microbiome profiling dataset

We downloaded the 16S microbiome profiling dataset (Study ID: 10483) generated by Sampson et al.^35^ from Qiita^36^. The dataset was in stored in Biological Observation Matrix (BIOM) format^45^ representing the absolute abundances of operational taxonomic units (OTUs) across samples. We also downloaded the corresponding metadata file describing the attributes of the samples from this study.

We performed quality assessments (QAs) for the datasets by examining the alpha- and beta- diversities as well as performing principal coordinate analysis (PCoA) to globally examine the samples in the OTU space. These QA steps were performed using the scikit-bio (version 0.5.4) Python package.

To perform differential abundance (DA) analysis, we employed the ANCOM^37^ with a one-way ANOVA test with a Bonferroni-corrected alpha of 0.1 as the rejection threshold, to identify DA microbes in mice transplanted with fecal samples from PD donors over healthy donors. The identified DA microbes were then used as the input for MSEA find enriched mammalian genes with known associations based on literatures. MSEA was performed using the “msea” Python package we developed following the statistical procedures described in previous sections.

### Reanalysis of the DSS-induced colitis microbiome profiling dataset

We downloaded the 16S microbiome profiling dataset (Study ID: 11123) generated by Nunberg et al.^39^ from Qiita^36^. The dataset was in stored in BIOM format^45^ representing the absolute abundances of OTUs across samples. We followed the same computational analysis pipeline as described for the PD dataset.

In addition, we performed pathway enrichment analysis for the genes identified by MSEA using Enrichr^38^. Concretely, the genes identified by MSEA using DA microbes in IL-1α-KO versus WT mice 8 days after DSS-administration were used as input for the Enrichr pathway analysis. The top 10 enriched human KEGG pathways were constructed to a network, where edges connect pathways with significant overlapping genes. Such significance in overlap was determined by Fisher’s exact test corrected p-value < 0.01. The network was then visualized using D3.js.

## Supplementary Information

Supplementary Information.

## Data Availability

All data generated or analysed during this study are included in this published article (and its Supplementary Information files).

## Code availability

The Python package “msea” is available at https://pypi.org/project/msea/. The documentation and tutorial can be found at https://msea.readthedocs.io/en/latest/.
