# Supplementary material for: Microbe-set enrichment analysis facilitates functional interpretation of microbiome profiling data
Source: Sci Rep. 2020 Dec 8;10:21466. doi: 10.1038/s41598-020-78511-y (PMC7722755; doi:10.1038/s41598-020-78511-y)
Supplement: Supplementary file 1 — Supplementary Information. [file 41598_2020_78511_MOESM1_ESM.pdf]

Supplementary information for:

Microbe-Set Enrichment Analysis Facilitates Functional Interpretation of Microbiome Profiling Data

Yan Kou[1], Xiaomin Xu[1], Zhengnong Zhu[1], Lei Dai[2,*], Yan Tan[1,*]

Affiliations

[1] Xbiome. Scientific Research Building, Room 907, Tsinghua High-Tech Park, Shenzhen, China

[2] CAS Key Laboratory of Quantitative Engineering Biology, Shenzhen Institute of Synthetic Biology , Shenzhen Institutes of Advanced Technology, Chinese Academy of Sciences, Shenzhen 518055, China

*** Corresponding authors**:

Lei Dai, lei.dai@siat.ac.cn

Yan Tan, yant@xbiome.com

Supplementary Figures

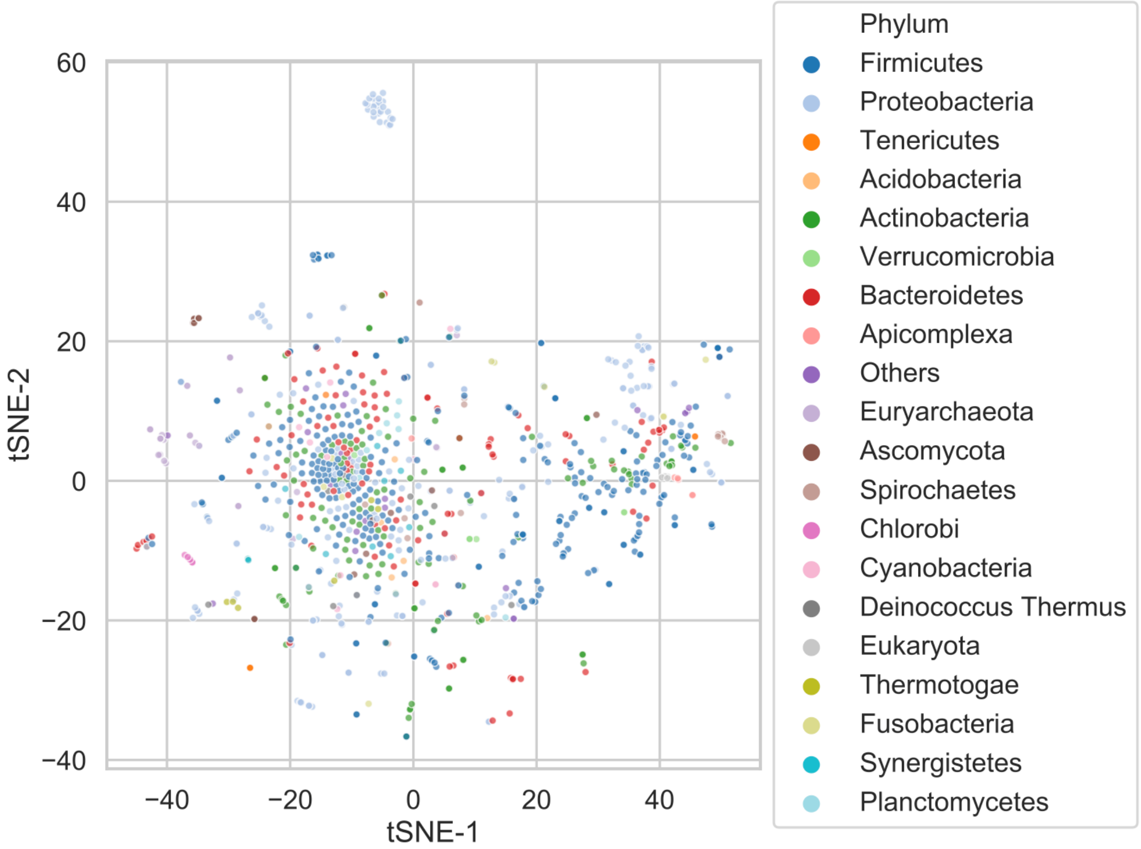

Figure S1. t-SNE visualization of the full normalized microbe-gene co-mentioning matrix derived from PubMed queries. The t-SNE was applied to the TF-IDF normalized microbe-gene co-mentioning matrix to calculate the 2-D coordinates for individual microbial genus or species. Each dot in the scatter plot represents a microbial genus or species, which is colored by their respective phylum based on Greengenes taxonomy.

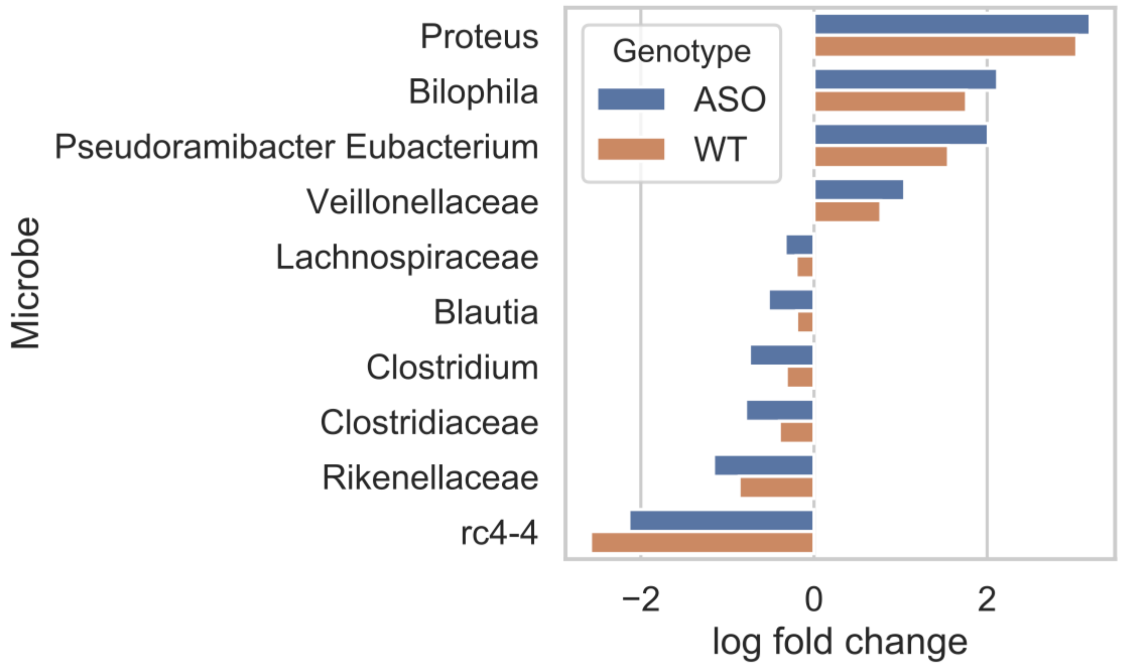

Figure S2. Microbes with differential abundance (DA) between mice transplanted with fecal samples from Parkinson's Disease (PD) patients and healthy donors. Germ-free mice from two genotypes: wild-type (WT) and alpha-synuclein-overexpressing (ASO) are colored in blue and orange, respectively. The bar plot shows the log2 fold change of the absolute abundances from the top 10 DA microbe genera.

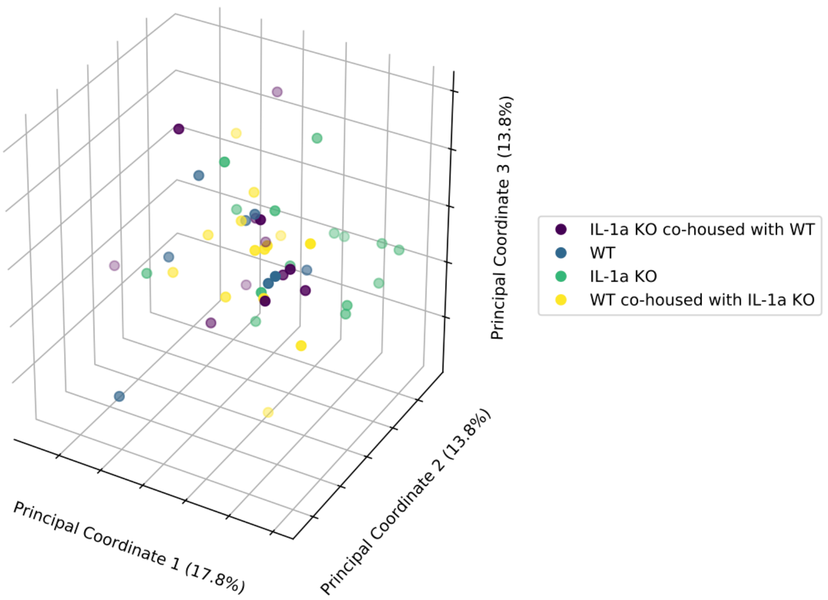

Figure S3. Scatter plots of fecal samples in case study 2 from Principal Coordinate Analysis (PCoA). PCoA was performed using unweighted UniFrac distances on the microbiome samples collected on day 8 after DSS-administration for IL-1α KO, WT, cohoused IL-1α KO, and cohoused WT mice.

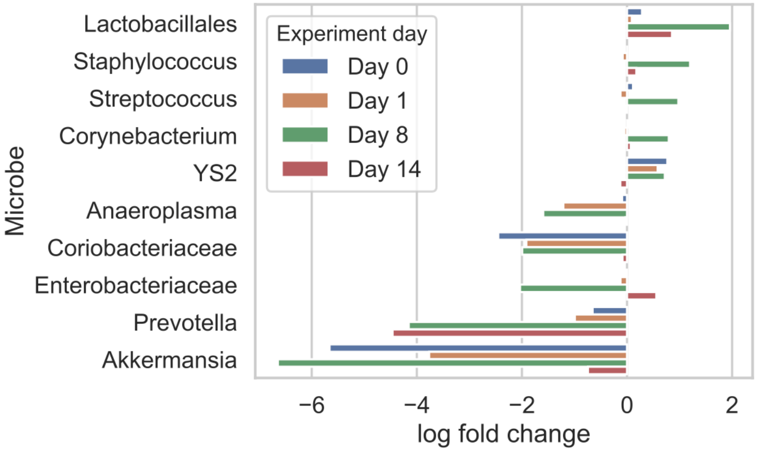

Figure S4. Microbes with differential abundance (DA) between WT and IL-1 α KO mice on day 0, 1, 8 and 14 after DSS-administration. The four time points are colored differently as indicated in the legend. The bar plot shows the log2 fold change of the absolute abundances from the top 10 DA microbe genera.

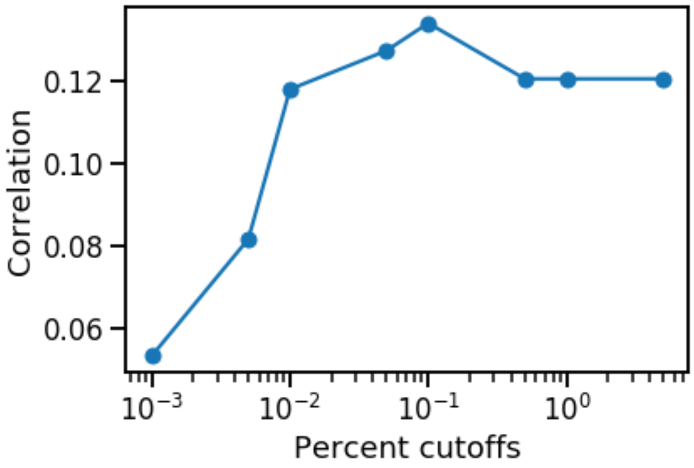

Figure S5. Quality assessment of microbe-set library constructed from PubMed query using different percentage cutoffs on the Jaccard index. The percentage cutoffs are plotted in log scale. Y-axis indicates the Pearson's correlation coefficient between the cosine distance vectors of the thresholded microbe-gene matrix of Jaccard index and the vector of the shortest path lengths on the taxonomy tree.

Supplementary Tables

Table S1. (Complementary to Table 3)

Gene	Disease	Odds ratio	p-value	q-value	z-score	Combined score	Number of shared microbes	Shared microbes
**SREBF1**	Non-alcoholic fatty liver disease	12.2	9.8E-08	2.0E-04	-3.95	63.80	11	Coprococcus, Prevotella, Escherichia, Ruminococcus, Lactobacillus,'Streptococcus, Dorea, Roseburia, Faecalibacterium, Blautia, Bacteroides
**LPL**	Non-alcoholic fatty liver disease	18.4	2.8E-07	2.9E-04	-1.35	20.43	8	Coprococcus, Escherichia, Ruminococcus, Lactobacillus, Streptococcus, Roseburia, Faecalibacterium, Blautia
**FABP4**	Hepatitis C	14.7	2.6E-07	5.4E-04	0.07	-1.07	9	Acinetobacter, Prevotella, Escherichia, Ruminococcus, Lactobacillus salivarius, Phascolarctobacterium, Lactobacillus, Streptococcus, Parabacteroides
**ATG16L1**	Crohn's Disease	3.9	2.2E-06	1.6E-03	-0.13	1.65	23	Odoribacter, Staphylococcus, Eubacterium, Alistipes, Mycobacterium, Bacteroidetes, Clostridium, Firmicutes, Lachnospiraceae, Bacteroides fragilis, Clostridium asparagiform, Bifidobacterium, Desulfovibrio, Enterobacteriaceae, Clostridium lactafifermentans, Faecalibacterium prausnitzii, Lactobacillus, Shigella, Clostridioides difficile, Citrobacter, Helicobacter, Faecalibacterium, Bacteroides
**CCL11**	Crohn's Disease	3.6	1.2E-06	1.6E-03	1.06	-14.42	27	Staphylococcus, Lactococcus, Pasteurellaceae, Holdemania, Clostridium, Pseudomonas, Enterococcus, Blautia, Escherichia, Ruminococcus, Actinobacillus, Lachnospiraceae, Bifidobacterium, Clostridium asparagiform, Haemophilus, Enterobacteriaceae, Clostridium lactafifermentans, Lactobacillus, Christensenella, Acinetobacter, Citrobacter, Anaerotruncus, Prevotella, Helicobacter, Bacillus, Parabacteroides, Proteobacteria
**FUT2**	Crohn's Disease	4.0	1.7E-06	1.6E-03	-2.26	30.03	23	Megasphaera, Turicibacter, Rikenellaceae, Bacteroidetes, Clostridium, Enterococcus, Ruminococcus, Lachnospiraceae, Bacteroides fragilis, Bifidobacterium, Clostridium asparagiform, Haemophilus, Lactobacillus, Clostridium lactafifermentans, Clostridioides difficile, Citrobacter, Prevotella, Helicobacter, Brachyspira, Faecalibacterium, Blautia, Bacteroides, Proteobacteria
**HCK**	Asthma	14.6	4.9E-06	1.7E-03	-1.85	22.60	7	Pseudomonas, Haemophilus, Gammaproteobacteria, Enterobacteriaceae, Streptococcus, Neisseria, Proteobacteria
**RAB14**	Asthma	15.9	3.1E-06	1.7E-03	0.98	-12.40	7	Moraxella, Haemophilus, Veillonella,

								Gammaproteobacteria, Enterobacteriaceae, Streptococcus, Proteobacteria
**TF**	Asthma	8.7	1.1E-05	1.7E-03	1.28	-14.60	9	Pseudomonas, Moraxella, Prevotella, Haemophilus, Enterobacteriaceae, Streptococcus, Neisseria, Clostridiaceae, Proteobacteria
**MAPK8**	Asthma	9.8	4.8E-06	1.7E-03	-1.52	18.64	9	Pseudomonas, Pseudomonadaceae, Haemophilus, Gammaproteobacteria, Lactobacillus, Enterobacteriaceae, Streptococcus, Neisseria, Moraxella

Table S2 (complementary to Figure 3) Top enriched human genes for DA microbes in PD versus healthy controls.

Gene	Odds ratio	P-value	q-value	Z-score	Combined score	Shared microbes
FUT2	16.2	5.2E-04	1.2E-01	-2.48	18.74	4
IL10	5.8	2.2E-03	1.3E-01	-2.75	16.75	7
FOXP3	6.6	4.6E-03	1.5E-01	-2.87	15.42	5
CYP7A1	14.0	3.5E-03	1.3E-01	-2.54	14.41	3
DEFB4A	8.5	4.3E-03	1.5E-01	-2.59	14.08	4
CCL2	7.1	1.7E-03	1.3E-01	-2.09	13.40	6
ANGPTL4	23.3	9.8E-04	1.2E-01	-1.84	12.73	3
FOS	6.5	2.4E-03	1.3E-01	-2.07	12.44	6
CCR2	10.6	2.1E-03	1.3E-01	-1.99	12.26	4
FFAR2	18.6	9.9E-03	1.8E-01	-2.53	11.70	2

Table S3 (complementary to Figure 4) Top enriched human genes for DA microbes in IL-1α-KO versus WT mice 8 days after DSS-administration.

Gene	Odds ratio	P-value	q-value	Z-score	Combined score	Shared microbes
SMAD7	14.2	3.1E-03	4.3E-02	-3.66	21.10	3
HLA-DPB1	23.1	1.0E-03	3.3E-02	-2.81	19.42	3
IL13	7.7	2.0E-03	3.9E-02	-3.12	19.40	5
IL4	7.5	2.2E-03	3.9E-02	-3.16	19.36	5
CCL11	11.5	1.3E-04	3.2E-02	-2.11	18.93	6
ELAVL1	30.8	5.3E-04	3.3E-02	-2.45	18.47	3
IL5	18.9	2.9E-04	3.3E-02	-2.17	17.66	4
SMAD3	11.7	1.3E-03	3.3E-02	-2.57	17.03	4
FOXO3	13.7	8.1E-04	3.3E-02	-2.31	16.45	4
GALM	18.5	1.7E-03	3.6E-02	-2.40	15.33	3
